# Both the Caspase CSP-1 and a Caspase-Independent Pathway Promote Programmed Cell Death in Parallel to the Canonical Pathway for Apoptosis in *Caenorhabditis elegans*

**DOI:** 10.1371/journal.pgen.1003341

**Published:** 2013-03-07

**Authors:** Daniel P. Denning, Victoria Hatch, H. Robert Horvitz

**Affiliations:** Howard Hughes Medical Institute and Department of Biology, Massachusetts Institute of Technology, Cambridge, Massachusetts, United States of America; The Rockefeller University, United States of America

## Abstract

Caspases are cysteine proteases that can drive apoptosis in metazoans and have critical functions in the elimination of cells during development, the maintenance of tissue homeostasis, and responses to cellular damage. Although a growing body of research suggests that programmed cell death can occur in the absence of caspases, mammalian studies of caspase-independent apoptosis are confounded by the existence of at least seven caspase homologs that can function redundantly to promote cell death. Caspase-independent programmed cell death is also thought to occur in the invertebrate nematode *Caenorhabditis elegans*. The *C. elegans* genome contains four caspase genes (*ced-3*, *csp-1*, *csp-2*, and *csp-3*), of which only *ced-3* has been demonstrated to promote apoptosis. Here, we show that CSP-1 is a pro-apoptotic caspase that promotes programmed cell death in a subset of cells fated to die during *C. elegans* embryogenesis. *csp-1* is expressed robustly in late pachytene nuclei of the germline and is required maternally for its role in embryonic programmed cell deaths. Unlike CED-3, CSP-1 is not regulated by the APAF-1 homolog CED-4 or the BCL-2 homolog CED-9, revealing that *csp-1* functions independently of the canonical genetic pathway for apoptosis. Previously we demonstrated that embryos lacking all four caspases can eliminate cells through an extrusion mechanism and that these cells are apoptotic. Extruded cells differ from cells that normally undergo programmed cell death not only by being extruded but also by not being engulfed by neighboring cells. In this study, we identify in *csp-3; csp-1; csp-2 ced-3* quadruple mutants apoptotic cell corpses that fully resemble wild-type cell corpses: these caspase-deficient cell corpses are morphologically apoptotic, are not extruded, and are internalized by engulfing cells. We conclude that both caspase-dependent and caspase-independent pathways promote apoptotic programmed cell death and the phagocytosis of cell corpses in parallel to the canonical apoptosis pathway involving CED-3 activation.

## Author Summary

Caspases are cysteine proteases that in many cases drive apoptosis, an evolutionarily conserved and highly stereotyped form of cellular suicide with functions in animal development and tissue maintenance. The dysregulation of apoptosis can contribute to diseases as diverse as cancer, autoimmunity, and neurodegeneration. Caspases are often thought to be required for, or even to define, apoptosis. Although there is evidence that apoptosis can occur in the absence of caspase activity, caspase-independence can be difficult to prove, as most animals have multiple caspases. The nematode *Caenorhabditis elegans* has four caspases, CED-3, CSP-1, CSP-2, and CSP-3. CED-3 has a well-established role in apoptosis, but less is known about the functions of the CSP caspases. In this study, we show that CSP-1 promotes apoptosis in the developing *C. elegans* embryo and that CSP-1 is regulated differently than its homolog CED-3. Furthermore, we show that apoptosis and the engulfment of dying cells can occur in mutants lacking all four caspases, proving that neither apoptosis nor cell-corpse engulfment require caspase function and that caspase-independent activities can contribute to apoptosis of some cells during animal development.

## Introduction

The elimination of unnecessary or dangerous cells is fundamental to development, tissue homeostasis and disease mitigation in multicellular organisms. The primary mechanism of cell elimination is apoptosis, a form of cell suicide that was originally defined by evolutionarily conserved morphological characteristics that include chromatin condensation, shrinkage of the cytoplasmic volume and membrane blebbing [1] and by biochemical features like phosphatidylserine exposure and DNA fragmentation [2], [3]. Apoptosis serves as a highly controlled mechanism for the removal and degradation of damaged or unnecessary cells, and blocking apoptosis can lead to catastrophic forms of cell death, such as necrosis, which can cause dangerous inflammatory responses [4]. The discovery of the CED-3 caspase as a cell-autonomous executioner of programmed cell death in the nematode *Caenorhabditis elegans* led to the paradigm that the caspase family of cysteine proteases drives apoptosis through the cleavage of substrate proteins at specific peptide sequences [5], [6]. Indeed, caspases have evolutionarily conserved roles in apoptosis throughout metazoa [7].

Despite the compelling causal link between caspases and apoptosis, a growing body of evidence indicates that apoptosis can occur in the absence of caspases [4]. For example, mouse cells lacking Apaf-1, an activator of the apical caspase Caspase-9, which in turn activates effector caspases, can undergo apoptosis in response to pro-apoptotic stimuli [8]. In the presence of caspase inhibitors, TNF (tumor necrosis factor) can induce a form of cell death termed necroptosis, which exhibits characteristics of both necrosis and apoptosis [4], [9]. The mitochondrial flavoprotein AIF (apoptosis-inducing factor) is thought to promote apoptotic cell death in mammals even in the presence of caspase inhibitors [10]. Furthermore, cell death with aspects of apoptotic morphology occurs in non-metazoans, including unicellular eukaryotes and prokaryotes, that lack clear caspase homologs [11], [12]. Thus, it is possible that apoptosis, as defined morphologically and biochemically, can occur in the absence of caspases.

A standard approach to assaying the caspase-dependence of apoptotic stimuli in tissue cell culture is through the pharmacological inhibition of caspases. However, it is difficult to prove that caspase activity is completely blocked in such experiments, and it is possible for caspase inhibitors to trigger non-apoptotic forms of cell death [13]. Studies of caspase-independent apoptosis in metazoans are also complicated by the existence of multiple caspases with potentially redundant functions in promoting cell death. The human genome, for example, encodes at least 10 caspase homologs, seven of which (*caspases-2*, *-3*, *-6*, *-7*, *-8*, *-9* and *-10*) have demonstrated roles in apoptosis [14]. The genome of *Drosophila melanogaster* encodes seven caspase homologs (*dcp-1*, *dronc*, *drice*, *dredd*, *decay*, *damm* and *strica*) [7], several of which are essential for organismal viability. The *C. elegans* genome encodes three caspase homologs (*csp-1*, *csp-2* and *csp-3*) in addition to *ced-3* [15]. Therefore, the use of mutant animals or cell lines deleted for one or two caspases might not eliminate all caspases expressed within a specific cell. Furthermore, since caspases have different substrate specificities [16], the use of a chemical substrate-competitive caspase inhibitor might not completely block all caspase activity. Ideally, experiments that test whether apoptosis can occur in the absence of caspases should be performed using mutant animals or cells that are genetically deleted of all caspase homologs. In this regard, *C. elegans* is an excellent animal for studies of caspase-independent programmed cell death, because: (1) there are several examples of *ced-3*-independent programmed cell death in *C. elegans* [17]–[19]; (2) mutants of *ced-3*, *csp-1*, *csp-2* and *csp-3* are viable [18]–[23]; and, (3) it is relatively easy to generate multiply mutant *C. elegans* strains.

The *ced-3* caspase gene is required for most programmed cell deaths that occur during *C. elegans* development [5], [20]. However, a small number of cells die in animals carrying null mutations of *ced-3*. The male-specific linker cell, which facilitates the connection of the vas deferens to the cloaca and then dies, undergoes a non-apoptotic cell death that bears morphological features (e.g., nuclear membrane crenellation) not seen with other *C. elegans* programmed cell deaths and that occurs in *ced-3* mutants as well as in animals doubly mutant for *ced-3* and *csp-1*, *csp-2* or *csp-3* [18], [20], [24]. We recently showed that a subset of cells fated to die in the *C. elegans* embryo are eliminated from *ced-3* mutants via a caspase-independent shedding mechanism [19]. Interestingly, the shed cells appear apoptotic, exhibiting chromatin condensation, TUNEL-reactive DNA degradation and phosphatidylserine exposure despite the absence of all four caspases. Unlike other apoptotic programmed cell deaths of *C. elegans*, the shed cells do not undergo phagocytosis by engulfing cells; instead, they are extruded from the developing embryo. By contrast, a small number of apoptotic cell corpses are visible in the heads of *ced-3* larvae [17]. Like other programmed cell deaths of *C. elegans*, these *ced-3*-independent cell corpses have a refractile appearance when viewed with Nomarski optics and are not extruded from the animal, suggesting that a *ced-3*-independent cell-killing activity contributes to these typical programmed cell deaths. The other caspase homologs, *csp-1*, *csp-2* and *csp-3*, are obvious candidates for driving this *ced-3*-independent cell-killing activity. However, it has recently been reported that *csp-2* and *csp-3* inhibit apoptosis in the germline and soma, respectively [22], [23].

To date, the *C. elegans* caspase homolog *csp-1* has no known function *in vivo*, including in apoptosis. An isoform of CSP-1 can cleave and possibly activate the CED-3 pro-protein *in vitro* [15]. We tested whether *csp-1* can promote or inhibit programmed cell death and whether it is regulated by the canonical programmed cell death pathway that activates *ced-3*. We found that *csp-1* encodes a pro-apoptotic caspase activity that promotes programmed cell death independently of the CED-3 caspase, CED-4 (the Apaf-1 homolog that activates CED-3), and CED-9 (a Bcl-2 family protein that negatively regulates CED-3 activation via inhibition of CED-4). Furthermore, we tested whether *csp-1*, *csp-2* and *csp-3* contribute to the *ced-3*-independent cell-killing activity that generates cell corpses in the heads of *ced-3* mutant larvae and found that these apoptotic cell deaths can occur in the complete absence of caspases. Thus, during *C. elegans* development programmed cell death followed by cell-corpse engulfment is achieved through three redundant pathways: (1) a *ced-3*-dependent pathway; (2) a *csp-1*-dependent pathway, which is not regulated by the canonical apoptosis pathway that controls *ced-3*; and, (3) a caspase-independent pathway.

## Results

### *csp-1* promotes the deaths of a subset of somatic cells fated to die

**Figure 1:**
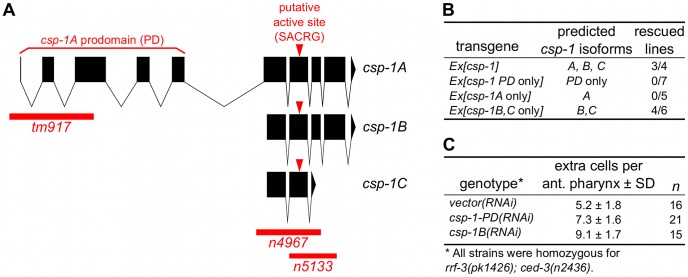
The B and/or C isoforms of *csp-1* promote programmed cell death. (A) Representations of the intron-exon organization of the three known *csp-1* mRNA isoforms (*A*, *B* and *C*). Red bars indicate the *csp-1* deletion alleles used in this study; arrowheads indicate the SACRG sequence that encodes the caspase active-site. The graphic was generated using the Intron-Exon Graphic Maker (N. Bhatla; www.wormweb.org). (B) Extrachromosomal arrays carrying a wild-type genomic fragment of the *csp-1* locus or a mutant version that expresses only the *B* or *C* isoforms can rescue the *csp-1(n4967)* mutant phenotype. The *csp-1 PD*-only transgene contains two nonsense mutations that encode early stop codons affecting the *B* and *C* mRNA isoforms; the *csp-1A*-only transgene contains a mutation that changes the *B*/*C* start codon to an alanine codon; and the *csp-1B/C*-only transgene contains two nonsense mutations that encode early stop codons affecting the *A* isoform. The *csp-1* transgenes were injected into *csp-1(n4967); ced-3(n2436)* animals, and the resulting independent lines were assayed for *csp-1* rescuing activity by counting the number of extra undead cells in the anterior pharynx. The transgenes and their constructions are described in detail in Materials and Methods, and the complete data for each transgenic line are provided in Table S2. (C) RNAi knockdown of *csp-1* phenocopies the *csp-1* mutations. dsRNAs targeting the *csp-1* pro-domain or the *csp-1B* isoform were *in vitro* transcribed and injected into the gonads of RNAi-sensitive *rrf-3(pk1426); ced-3(n2436)* adult hermaphrodites. Progeny of the injected adults were assayed for extra undead cells in the anterior pharynx. PD, prodomain.

The *C. elegans* genes *csp-1*, *csp-2* and *csp-3* are paralogs of the pro-apoptotic *ced-3* caspase gene [15], which is required for most programmed cell deaths in the worm [5], [20]. Given the conserved role of caspases in apoptosis, we tested *csp-1*, *csp-2* and *csp-3* for roles – both pro- and anti-apoptotic – in programmed cell death. We used mutations of *csp-1* (*n4967* and *n5133*) and *csp-2* (*n4871*) that completely remove the genomic sequences encoding their respective predicted caspase active sites (SACRG in the CSP-1 protein, and VCCRG in the CSP-2 protein) and therefore eliminate any potential caspase activity encoded by these genes (ref. [19]; Figure 1A). *csp-3* lacks a caspase active site (ref. [15], [22]; Figure 1A); we used the *csp-3* deletion mutation *n4872*, which is likely a null allele [19].

Recently, it was reported that mutations in *csp-2* and *csp-3* cause ectopic cell deaths in the germline and soma, respectively, and hence that *csp-2* and *csp-3* inhibit apoptosis [22], [23]. We therefore tested whether *csp-1* mutants have ectopic cell deaths indicative of a loss of anti-apoptotic function. Using Nomarski optics and a P*_mec-3_::gfp* transgene that expresses GFP in the six touch neurons (AVM, two ALM, PVM and two PLM neurons) in addition to the FLP and PVD neurons, we examined *csp-1* mutants for missing cells that normally survive. We observed that *csp-1(n4967)* mutants contained a full complement of touch neurons and pharyngeal cells (Table S1). We also noted that *csp-1(n4967)* failed to cause ectopic cell deaths in sensitized animals carrying the loss-of-function mutation *n2812* in the anti-apoptotic gene *ced-9*, a homolog of human *BCL2* (Table S1; data not shown). These results indicate that *csp-1* does not have an obvious anti-apoptotic function in the soma. Consistent with a previous report that *csp-2* does not affect somatic cells [23], a mutation in *csp-2* did not cause ectopic cell deaths in the somatic cells we examined (Table S1). However, we failed to observe the ectopic cell deaths in *csp-3* mutants previously reported [22]. Ectopic somatic cell deaths have also been noted in animals with loss-of-function mutations in *ced-9* [25] or *tat-1* [26], [27], which encodes an aminophospholipid translocase required for the asymmetric distribution of phosphatidylserine on the inner leaflet of the plasma membrane. As expected, we found that *ced-9* mutant larvae were missing pharyngeal cells and many touch neurons: more than 80% of PLM neurons were not present in *ced-9(n2812)* larvae (Table S1). However, we failed to detect the previously reported ectopic cell-death defect of *tat-1* mutants (ref. [26], [27]; Table S1); we used the same deletion alleles for *csp-3* and *tat-1* and assayed the same cells that had been characterized in the previous studies.

To determine whether the *C. elegans* caspase homologs *csp-1*, *csp-2* or *csp-3* promote programmed cell death in the soma, we examined animals carrying *csp* deletion mutations for extra cells that failed to undergo programmed cell death in the anterior pharynx. As many as 16 extra cells can be counted in the anterior pharynges of mutants with strong defects in programmed cell death, e.g., *ced-3(n3692)* (ref. [28]; Table 1). Single mutations in *csp-1*, *csp-2* or *csp-3* failed to cause detectable defects in programmed cell death (Table 1; data not shown). However, we observed that mutations in *csp-1* (but not *csp-2* or *csp-3*) caused the survival of pharyngeal cells in sensitized strains carrying a weak mutation in the caspase gene *ced-3* (Table 1). The partial loss-of-function *ced-3* mutations *n2427* and *n2436* cause slight and intermediate defects in apoptosis, respectively (ref [17]; Table 1; data not shown). The *n4967* and *n5133* mutations, both of which delete the putative active site of CSP-1 (Figure 1A), enhanced the cell-death defects of *ced-3(n2427)* and *ced-3(n2436)* mutants, increasing the number of extra cells in their anterior pharynges on average by 1.4 and 2.4 cells, respectively (Table 1). These results are consistent with our RNAi experiments in which *csp-1B* dsRNA (which likely inactivated all *csp-1* transcripts) was injected into the gonads of *rrf-3(pk1426); ced-3(n2436)* animals and caused an enhanced cell-death defect in their progeny (Figure 1C); we used the *rrf-3* mutation to increase sensitivity to RNAi [29]. The cell-death defect conferred by the *csp-1(n4967)* mutation was rescued by extrachromosomal arrays carrying a 9 kb genomic *csp-1* fragment that included the entire *csp-1* coding region, 1.5 kb of genomic sequence 5′ of the *csp-1A* translational start codon and 3.5 kb of genomic sequence 3′ of the *csp-1A/B* translational stop codon (Figure 1B; Table S2). These results demonstrate that *csp-1* encodes a detectable cell-killing activity that contributes to programmed cell death in *C. elegans*. Mutation of *csp-2* and/or *csp-3* neither enhanced nor suppressed the cell-death defects of strains mutant for *csp-1* and/or *ced-3* (Table 1; Table S3), suggesting that *csp-1* and *ced-3* are the only *C. elegans* caspase genes with functions in somatic programmed cell deaths.

**Table 1:** The caspase homolog *csp-1* promotes programmed cell death in the *C. elegans* anterior pharynx.

genotype	extra cells per anterior pharynx ± SD	*n*	*p* value
**The deletion of *csp-1*, *csp-2* or *csp-3* alone does not cause a defect in programmed cell death.**			
wild-type^1^	0.1±0.3	14	-
*ced-3(n3692)* 1	11.3±1.1	14	<0.00001
*csp-1(n4967)*	0.3±0.4	16	n.s.
*csp-1(n5133)*	0.1±0.2	19	n.s.
*csp-1(tm917)*	0.1±0.3	16	n.s.
*csp-2(n4871)* 1	0.2±0.4	12	n.s.
*csp-3(n4872)* 1	0.3±0.6	21	n.s.
**Deletion of *csp-1*, but not *csp-2* or *csp-3*, enhances the defects in programmed cell death caused by partial loss-of-function alleles of *ced-3* and *ced-4*.**			
*ced-3(n2427)* 1	1.7±1.2	22	-
*csp-1(n4967); ced-3(n2427)* 2	3.0±1.3	38	0.0001
*csp-1(n5133); ced-3(n2427)*	3.2±1.5	21	0.0008
*csp-1(tm917); ced-3(n2427)* 2	2.6±1.3	46	0.006
*csp-2(n4871) ced-3(n2427)* 1	1.0±0.8	20	n.s.
*csp-3(n4872); ced-3(n2427)* 3	1.5±1.2	16	n.s.
*csp-3; csp-2 ced-3(n2427)* 1	1.9±1.5	18	-
*csp-3; csp-1(n4967); csp-2 ced-3(n2427)*	3.2±1.2	17	0.008
			
*ced-3(n2436)* 1	6.2±1.4	37	-
*csp-1(n4967); ced-3(n2436)*	8.6±1.6	29	<0.00001
*csp-1(n5133); ced-3(n2436)*	8.7±1.5	19	<0.00001
*csp-1(tm917); ced-3(n2436)* 2	7.4±1.6	52	<0.00001
*csp-2(n4871) ced-3(n2436)* 1	5.4±1.0	16	n.s.
*csp-3(n4872); ced-3(n2436)* 1	5.6±1.5	15	n.s.
*csp-3; csp-2 ced-3(n2436)* 1	4.9±1.7	16	-
*csp-3; csp-1(n4967); csp-2 ced-3(n2436)*	8.2±1.4	16	<0.00001
			
*ced-4(n3158)* 1	5.0±2.6	29	-
*csp-1(n4967); ced-4(n3158)* 1	6.5±2.7	32	0.03

1 Homozygous for the integrated transgene *nIs106[P_lin-11_::gfp]*.

2 Includes animals that were either homozygous for *unc-75(+)* or *unc-75(e950)*.

3 Homozygous for *dpy-20(e1282ts)*.

For the statistical comparisons between *ced-3(n2427)* or *ced-3(n2436)* and double mutants with each *csp* allele, *p* values were considered significant if less than 0.01 to correct for multiple comparisons.

The development of the anterior part of the *C. elegans* pharynx involves 16 programmed cell deaths, all of which appear to be sensitive to *ced-3* [17], [28], [30]. To test whether specific pharyngeal programmed cell deaths required *csp-1*, we used GFP reporters to visualize the survival of cells fated to die, specifically the sister cells of the M4 and NSM neurons. *csp-1* was partially required in *ced-3(n2427)* or *ced-3(n2436)* sensitized strains for the death of the M4 sister cell (Table S4); by contrast, mutation of *csp-1* did not affect the cell deaths of the sister cells of the NSM neurons (data not shown). Likewise, *csp-1* did not appear to function in the postembryonic programmed cell deaths of the ventral cord or postdeirid sensilla (Table S4). We conclude that *csp-1* promotes cell death in a subset of cells fated to die during *C. elegans* development.

### The *csp-1B* and/or *C* isoforms are required for the cell-killing activity of *csp-1*

**Figure 2:**
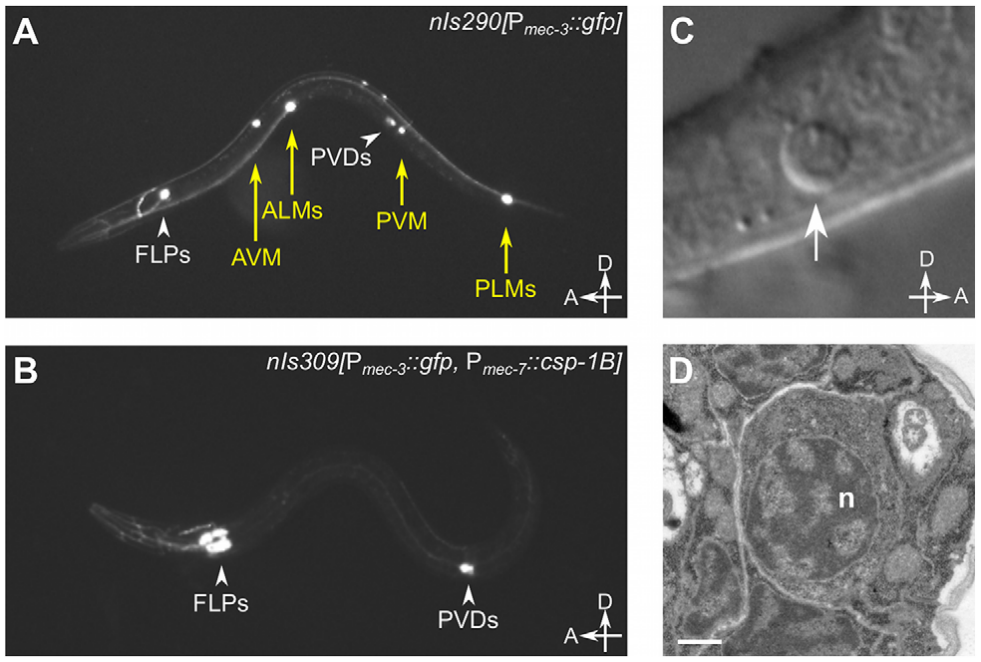
*csp-1B* overexpression induces ectopic cell deaths. (A) Fluorescence image of a transgenic *nIs290[*P*_mec-3_::gfp]* larva expressing GFP from the *mec-3* promoter in the touch neurons (AVM, ALMs, PVM and PLMs, yellow arrows). *mec-3* is also expressed in the FLP and PVD neurons (white arrowheads). (B) Fluorescence image of a transgenic *nIs309[*P*_mec-7_::csp-1B*, P*_mec-3_::gfp]* larva expressing CSP-1B from the *mec-7* promoter (which is expressed in the AVM, ALM, PVM and PLM neurons) and GFP from the *mec-3* promoter. Note the absence of touch neurons. (C) Nomarski differential interference contrast (DIC) image of a refractile PLM cell corpse (arrow) in a *ced-1(e1735); ced-4(n1162); nIs309* L1 larva. (D) Transmission electron microscopic image of the cell corpse in (C). “n”, nucleus of the cell corpse; scale bar, 0.5 microns.

The *csp-1* locus produces three known mRNA isoforms [15], all of which include the sequence that encodes the presumptive caspase active site (Figure 1A). The *csp-1A* transcript contains a long prodomain not present in the other transcripts, and it uses an alternative start site that is 3 kb 5′ to the start site of the *csp-1B* and *csp-1C* isoforms. To determine which isoforms are required for the cell-killing activity of *csp-1*, we peformed experiments in which the *csp-1* rescuing transgene was mutated to express: (1) the *A* isoform only, (2) the *B* and *C* isoforms only, or (3) a truncated version of *csp-1A* including only the prodomain (PD). Extrachromosomal arrays engineered to express only *csp-1-PD* or the *csp-1A* isoform failed to rescue the cell-death defect of *csp-1(n4967)* mutants (Figure 1B; Table S2). By contrast, a *csp-1* transgene lacking the *csp-1A* translation start codon and predicted to express only the *csp-1B* and *csp-1C* transcripts rescued the *csp-1(n4967)* defect in programmed cell death (Figure 1B; Table S2). Consistent with these results, transgenes expressing the *csp-1B* cDNA, but not the *csp-1A* cDNA, under the control of the *mec-7* promoter efficiently killed touch neurons (Figure 2A–2B; Table 2; data not shown); we also expresed the *csp-1C* cDNA under the control of the *mec-7* promoter and failed to observe killing of the touch neurons (data not shown). Ectopic expression of *csp-1B* from the *ser-2d* and *flp-15* promoters killed the OLL and I2 neurons, respectively (ref. [31]; N. Bhatla and H.R. Horvitz, unpublished results). However, we noted that *tm917*, a *csp-1* allele that deletes coding regions of only the *csp-1A* transcript, enhanced significantly (albeit weakly) the cell-death defects of *ced-3(n2427)* and *ced-3(n2436)* mutants, increasing the number of extra cells in their anterior pharynges by 0.9 and 1.2 cells, respectively (Table 1). dsRNA targetting the *csp-1A* prodomain (*csp-1-PD*) caused a similar slight enhancement of the cell-death defect of *ced-3(n2436)* mutants (Figure 1C), suggesting that, in addition to the more robust cell-killing activity of the *csp-1B* transcript, *csp-1A* might have a weak cell-killing function.

**Table 2:** Ectopic expression of *csp-1B* from the *mec-7* promoter can kill touch neurons, and this killing activity requires the conserved cysteine in the putative caspase active site.

		% survival			
genotype*	*n*	AVM	ALML/R	PVM	PLML/R
wild-type *[nIs290]*	59	100	100	100	100
P*_mec-7_::csp-1B* Line #1 *[nIs307]*	52	71	61	40	13
P*_mec-7_::csp-1B* Line #2 *[nIs308]*	41	49	32	27	16
P*_mec-7_::csp-1B* Line #3 *[nIs309]*	60	2	1	3	0
P*_mec-7_::csp-1B(C138S)* Line #1 *[nIs368]*	23	100	100	100	98
P*_mec-7_::csp-1B(C138S)* Line #2 *[nIs369]*	23	100	100	100	87
P*_mec-7_::csp-1B(C138S)* Line #3 *[nIs370]*	25	100	100	100	100

*n*, number of animals assayed; for each animal, six touch neurons (AVM, ALML, ALMR, PVM, PLML and PLMR) were scored for survival using the P*_mec-3_::gfp* reporter transgene.

* Each strain contained the transgene P*_mec-3_::gfp*, which expressed GFP in the FLP, AVM, ALM, PVM, PVD and PLM neurons.

### *csp-1B* encodes a pro-apoptotic caspase

The proteolytic activity of caspases requires an active-site cysteine. Previously, it was shown that the CSP-1B protein can proteolytically process CED-3 *in vitro* and that this enzymatic activity required the active-site (SACRG) cysteine of CSP-1B, C138 [15]. We tested *in vivo* whether C138 was necessary by assaying the touch neuron-killing activity of mutant P*_mec-7_::csp-1B* transgenes in which C138 was changed to a serine. We observed that the ectopic cell deaths were entirely dependent on the caspase active site (Table 2). Thus, *csp-1B* promotes cell death via caspase activity. The cell deaths induced by a P*_mec-7_::csp-1B* transgene resulted in cell corpses with apoptotic characteristics (Figure 2C–2D). When observed with Nomarski optics, the *csp-1B*-induced cell deaths exhibited a refractile button-like appearance (Figure 2C) similar to that of developmental programmed cell deaths. Transmission electron micrographs of the cell corpses showed some contraction of the cytoplasmic volume and considerable condensation of the nuclear chromatin (Figure 2D), which are general characteristics of apoptotic cells, including those generated by *ced-3* cell-killing transgenes (ref. [32]; data not shown). We conclude that *csp-1B* encodes a functional caspase that promotes programmed cell deaths with apoptotic morphology.

### *csp-1B* cell-killing activity is independent of *ced-9* and *ced-4*

CED-3, like most caspases, is expressed as an inactive zymogen with an inhibitory N-terminal prodomain. *Trans*-auto-proteolysis of the CED-3 pro-protein at two aspartate residues removes the pro-domain and yields two subunits that form the active caspase [33]. CED-3 auto-activation is dependent on its prodomain and is facilitated by the association of two CED-3 pro-proteins within an octameric complex formed with the Apaf-1 homolog CED-4 [34]–[36]. Under normal cellular conditions, CED-4 is sequestered by CED-9 at mitochondria through a direct protein-protein interaction [37]–[39]. In response to upstream pro-apoptotic signals and the consequent expression of the BH3-domain-only protein EGL-1, which binds to and inhibits CED-9 [40], CED-4 is released from CED-9 and translocates to the nuclear periphery [37], [41], where it facilitates CED-3 activation [38]. Thus, the activation of CED-3 is controlled by an apoptosis pathway involving a BH3-domain-only protein, a member of the Bcl-2 family of apoptosis regulators, and a homolog of the apoptosome complex protein Apaf-1. The basic elements of this apoptosis pathway are evolutionarily conserved in mammals and are responsible for the activation of caspases in response to cell-intrinsic apoptotic stimuli [7].

**Figure 3:**
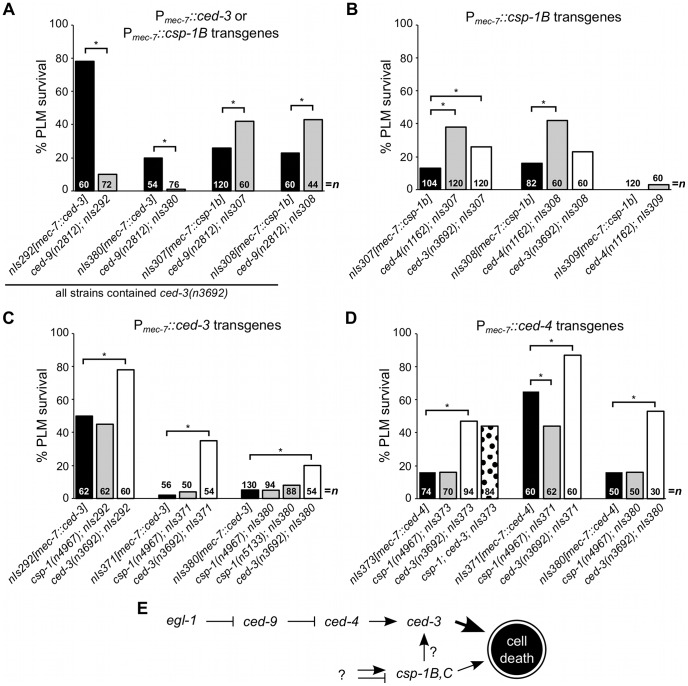
*csp-1B* cell-killing activity is not regulated by the canonical programmed cell death pathway. (A–D) The percentages of PLM cells that survive in strains carrying P*_mec-7_::ced-3*, P*_mec-7_::csp-1B* or P*_mec-7_::ced-4* transgenes. (A) *ced-9* protects against *ced-3*- but not *csp-1B*-cell-killing transgenes. (B) The cell-killing activity of *csp-1B* transgenes is mostly independent of *ced-3* and *ced-4*. The cell-killing activities of (C) *ced-3* and (D) *ced-4* transgenes do not require *csp-1*. PLM survival was scored based on the presence of GFP expressed from the *mec-3* promoter. Asterisks indicate *p*<0.05 in a Fisher's exact test. All strains in (A) contained *ced-3(n3692)*. (E) A model depicting the genetic pathways regulating the caspase genes *csp-1* and *ced-3* (see text).

Consistent with the role of *ced-9* in negatively regulating *ced-3* activation, it was previously shown that null mutations of *ced-9* enhance the touch neuron-killing activities of P*_mec-7_::ced-3* transgenes [32]. (These experiments were performed using a *ced-3(null)* background to suppress the *ced-3*-dependent inviability of *ced-9(null)* animals.) Furthermore, this enhancement is dependent on *ced-4* [32], indicating that the absence of CED-9 activates endogenous CED-4 within the touch neurons and that CED-4 activation elevates CED-3 activity. Unlike the CED-3 zymogen, CSP-1B lacks a long prodomain, suggesting that it might be activated via an alternative mechanism (i.e., independently of CED-4 and CED-9). To determine whether these canonical apoptosis regulators control CSP-1B activation, we introduced the *ced-9(n2812)* mutation into *ced-3(n3692)* strains carrying P*_mec-7_::csp-1B* transgenes and assessed the effect of this *ced-9* null mutation on PLM survival. In contrast to its effects on P*_mec-7_::ced-3*–mediated PLM killing, *ced-9(n2812)* failed to enhance PLM killing in P*_mec-7_::csp-1B* strains with a *ced-3(n3692)* mutant background (Figure 3A). Instead, *ced-9(n2812)* partially suppressed *csp-1B*-mediated PLM death (Figure 3A). CED-9 has a poorly understood pro-apoptotic activity in addition to its anti-apoptotic role in CED-4 inhibition [42], and it is possible that this *ced-9* pro-apoptotic activity contributed to the deaths of cells expressing ectopic CSP-1B. Nevertheless, our results indicate that *csp-1B*-mediated cell killing, unlike *ced-3*-mediated cell killing, is not negatively regulated by *ced-9* and suggest that CSP-1B is activated independently of CED-9.

We also observed that the expression of a P*_mec-7_::csp-1A* transgene in *ced-3(null)* mutant strains failed to cause PLM cell death, even in a *ced-9(null)* background (Figure S1). These results suggest that the CSP-1A isoform (which contains a long prodomain similar to that of CED-3) does not promote programmed cell death, even in the absence of the anti-apoptotic protein CED-9. A role for *csp-1A* in cell death cannot be excluded entirely, as it is possible that endogenous CSP-1A requires a co-factor not present in the touch neurons to mediate cell killing.

Since CSP-1B can proteolytically cleave CED-3 *in vitro* [15], we tested whether the *csp-1B* cell-killing activing requires the endogenous *ced-3* and *ced-4* genes. The *ced-3(n3692)* and *ced-4(n1162)* mutations weakly suppressed *csp-1B*-mediated PLM death (Figure 3B), and it is possible that the endogenous *csp-1* can in part promote programmed cell death through *ced-3*. Nonetheless, most *csp-1B* cell-killing activity was independent of *ced-4* and *ced-3* (Figure 3B). Loss of endogenous *csp-1* failed to suppress PLM death in strains carrying P*_mec-7_::ced-3* or P*_mec-7_::ced-4* transgenes (Figure 3C–3D). Together, our results are consistent with a model in which *csp-1B* promotes programmed cell death at least mostly independently of and in parallel to the canonical apoptosis pathway (Figure 3E).

### *csp-1* expression in the maternal germline contributes to embryonic programmed cell death

**Figure 4:**
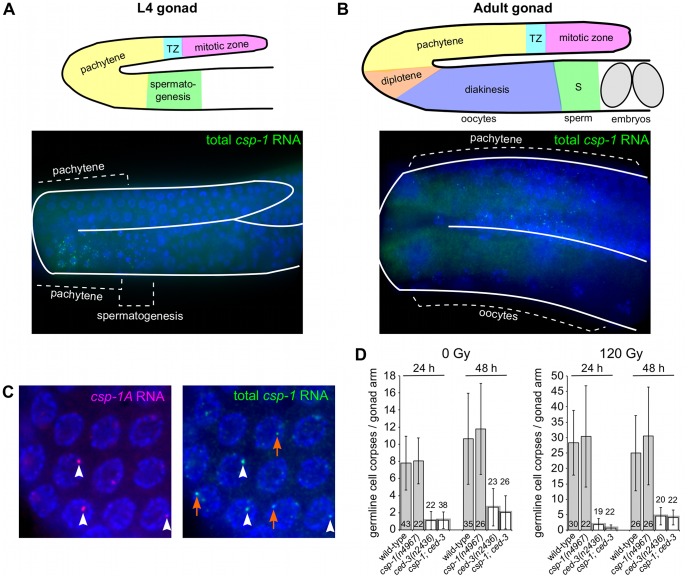
*csp-1* is expressed in late pachytene cells of the L4 and adult hermaphrodite germline. Fluorescence *in situ* hybridization images of gonad arms of (A) an L4 hermaphrodite and (B) an adult hermaphrodite hybridized with Cy5-labelled probes complementary to *csp-1B*. The Cy5-labelled probes are visible as green puncta; the gonads are outlined in white. A schematic representation is shown above each micrograph. (C) Fluorescence *in situ* hybridization images of an adult hermaphrodite gonad hybridized with ALEXA594-labelled probes (red puncta) complementary to the region of *csp-1A* that encodes the prodomain (*csp-1A*) and Cy5-labelled *csp-1B* probes (green puncta) that hybridize to all *csp-1* transcript isoforms (total *csp-1*). White arrowheads indicate *csp-1A*-specific puncta; orange arrows indicate *csp-1B*-specific puncta, which are recognized strongly by the total *csp-1* probes but only weakly by the *csp-1A*-specific probes. (D) The number of CED-1::GFP-positive apoptotic cells in the gonads of caspase mutants exposed to 0 Gy and 120 Gy of ionizing radiation at the L4 larval stage. The strains were scored at 24 hrs and 48 hrs post L4-stage. Error bars indicate standard deviations.

To determine which *C. elegans* cells express *csp-1*, we directly visualized endogenous *csp-1* transcripts via fluorescence *in situ* hybridization (FISH) experiments using Cy5- and ALEXA-labelled probes complementary to the *csp-1B* transcript (i.e., targeted to all *csp-1* transcripts) or to the *csp-1A* prodomain (specific to the *csp-1A* trancript). To our surprise, *csp-1* mRNA was not detectable in the somatic cells of wild-type or *egl-1(n1084 n3082)* mutant embryos, larvae or adult hermaphrodites (data not shown). By contrast, *csp-1* transcripts were present in the germlines of L4-stage larval and adult hermaphrodites (Figure 4A–4B). This expression was restricted to the late pachytene stage of meiosis I in both L4 larval gonads (in pachytene nuclei adjacent to differentiating sperm) and adult gonads (in pachytene nuclei adjacent to the bend of the gonad arm) (Figure 4A–4B). Both *csp-1A* and *csp-1B/C* transcripts were expressed in the adult pachytene germ cells, as indicated by the presence of FISH foci recognized by the *csp-1A* prodomain probes and foci recognized primarily by the *csp-1B* probes and only weakly by the *csp-1A* probes (Figure 4C). Stochastic and ionizing radiation (IR)-induced germline cell deaths occur during the late pachytene stage of oocyte development in adult gonads [43], [44]. However, *csp-1* (unlike *ced-3*) was not required for either stochastic or IR-induced germline apoptosis, even in *ced-3(n2436)* strains sensitized for defects in germ-cell death (Figure 4D). In these experiments, apoptotic germ cells were identified using a transgene that expresses a functional GFP::CED-1 fusion protein that envelopes dying cells engulfed by the gonadal sheath [45], [46]. We also failed to detect differences in either stochastic or IR-induced germline cell death between *csp-1* mutants and wild-type animals in experiments in which apoptotic germ cells were quantified by acridine orange staining or by direct observation of their refractile morphology using Nomarski optics (data not shown). We also noted that the level of *csp-1* transcript expression in the germline (as determined by FISH) was not affected by either ionizing radiation or by mutation of *egl-1* or *ced-3* (data not shown).

Since we detected *csp-1* expression in the adult germline but not in somatic cells of the embryo, we tested whether maternally supplied *csp-1* transcript was necessary for the zygotic function of *csp-1* in programmed cell death. Indeed, in sensitized genetic backgrounds (*ced-3(n2427)* and *ced-3(n2436)*), *csp-1(+)* progeny of *csp-1(n4967)* hermaphrodites (M−Z+ animals) had more undead pharyngeal cells than the *csp-1(+)* progeny of *csp-1(+)* hermaphrodites (M+Z+ animals) or the *csp-1(n4967)* progeny of *csp-1(+)* hermaphrodites (M+Z− animals) (Table 3). Thus, *csp-1* expressed in the maternal germline is necessary for the *csp-1* pro-apoptotic activity in embryonic programmed cell deaths. Given that we could not detect *csp-1* expression in either embryos or larvae, it is therefore not surprising that the postembryonic programmed cell deaths of the ventral cord and postdeirid sensilla were unaffected by mutation of *csp-1* (Table S4).

**Table 3:** *csp-1* is maternally required for programmed cell deaths that occur embryonically in the presumptive anterior pharynx.

zygotic genotype	maternal genotype	extra cells per ant. pharynx ± SD	*n*	*p* value
*ced-3(n2427)* a	*ced-3(n2427)*	1.0±0.9	20	-
*csp-1(n4967)/+; ced-3(n2427)* b	*csp-1(n4967); ced-3(n2427)*	3.2±1.2	20	<0.0001
*csp-1(n4967)/+; ced-3(n2427)* a	*ced-3(n2427)*	1.7±1.0	20	0.018
*ced-3(n2436)* a	*ced-3(n2436)*	6.2±1.2	18	-
*csp-1(n4967)/+; ced-3(n2436)* b	*csp-1(n4967); ced-3(n2436)*	8.0±1.8	18	0.002
*csp-1(n4967)/+; ced-3(n2436)* a	*ced-3(n2436)*	6.3±1.3	18	n.s.

a Heterozygous for *unc-30(e191)/+*.

b Heterozygous for *unc-75(e950)/+*.

### Programmed cell deaths occur in animals completely lacking all caspase genes

Most programmed cell deaths in *C. elegans* require *ced-3* [20]. However, some cells die in mutants completely lacking *ced-3*. We previously reported that a subset of cells fated to die can be eliminated from *ced-3* mutant embryos via a cell-shedding mechansm [19]. In that study, we noted that cell shedding from *ced-3* mutants occurs independently of *csp-1*, *csp-2* and *csp-3*: quadruple mutants lacking all four caspases also generate shed cells, indicating that cell elimination by this mechanism is completely caspase-independent [19]. Like most programmed cell deaths, the cells generated by caspase-independent extrusion are apoptotic in appearance. However, unlike caspase-dependent cell corpses, shed cells do not undergo phagocytosis by engulfing cells. The death of the male linker cell, which also occurs independently of *ced-3*, is non-apoptotic and requires the heterochronic protein LIN-29, its binding partner MAB-10 [47], and the polyglutamine repeat protein PQN-41 (ref. [18], [24]; Table S5). Previously it was shown that this cell death occurs in double-mutant males in which *ced-3* and an additional *csp* gene (*csp-1*, *csp-2* or *csp-3*) were inactivated [18]. We have now examined males lacking all four caspases and observed that the linker cell died in 100% of *csp-3; csp-1; csp-2 ced-3* mutants (Table S5). The *csp-3; csp-1; csp-2 ced-3* quadruple mutants were viable and fertile. Thus, both zygotic and maternal caspase contributions were eliminated. Our results therefore confirm that this cell death is indeed completely caspase-independent.

In addition, cell corpses are visible in the heads of larvae carrying null alleles of *ced-3* (ref. [17]; Table 4). All programmed cell deaths in the developing heads of wild-type animals occur embryonically and are engulfed and degraded prior to hatching (ref. [30], [48]; Table 4). To detect *ced-3*-independent programmed cell deaths in larval heads, we used mutations (e.g., *ced-1(e1735)*, *ced-6(n2095)* or *ced-7(n1996)*) that cause defects in cell-corpse engulfment and result in the persistence of many embryonic cell corpses into larval stages (ref. [49], [50]; Table 4). Like most wild-type cell corpses, the *ced-3*-independent cell corpses were refractile in appearance as observed with Nomarski optics and were not extruded from the animal (data not shown). We also observed that larvae mutant for *ced-4* or *egl-1* contained similar cell corpses, demonstrating that their generation does not require the canonical pro-apoptotic pathway that mediates most programmed cell deaths (Table 4).

**Table 4:** Cell deaths occur in the absence of all *C. elegans* caspase genes.

genotype	*n*	% with ≥1 corpse	corpses per head ± SD
*ced-1(e1735)*	23	100	21.3±5.3
*ced-1; ced-3(n3692)*	49	27	0.3±0.5
*ced-1; ced-4(n1162)*	25	36	0.4±0.6
*ced-1; egl-1(n1084 n3082)*	26	50	0.6±0.7
*ced-1; csp-1(n4967)*	29	100	23.4±4.7
*ced-1; csp-2(n4871)*	24	100	20.7±5.6
*ced-1; csp-1(n4967); ced-3(n3692)*	30	27	0.3±0.5
*ced-1; csp-1(n4967); csp-2(n4871) ced-3(n3692)*	24	21	0.2±0.4
*ced-7(n1996)*	21	100	30.1±4.4
*ced-7; ced-3(n3692)*	22	23	0.3±0.6
*csp-1(n4967); ced-7*	19	100	30.3±4.3
*csp-1(n4967); ced-7; ced-3(n3692)*	19	37	0.4±0.5
*csp-3(n4872); csp-1(n4967); ced-7; csp-2(n4871) ced-3(n3692)*	32	34	0.4±0.6
*ced-6(n2095)*	24	100	19.8±4.3
*csp-3(n4872); csp-1(n4967); ced-6; csp-2(n4871) ced-3(n3692)*	36	39	0.4±0.6
wild-type	28	0	0.0±0.0
*ced-3(n3692)*	43	14	0.1±0.4
*ced-3(n2452)*	27	41	0.4±0.5
*csp-3(n4872); csp-1(n4967); csp-2(n4871) ced-3(n3692)*	25	16	0.2±0.4
*csp-3(n4872); csp-1(n4967); csp-2(n4871) ced-3(n2452)*	34	26	0.3±0.4

The number of refractile cell corpses per head was counted in L1 larvae within one hour of hatching.

We tested whether the small number of cell corpses visible in *ced-3* larval heads are generated by the other *C. elegans* caspase genes and found that all double, triple and quadruple caspase mutants that we examined contained a small number of refractile corpses (Table 4). For example, 39% of *csp-3; csp-1; ced-6; csp-2 ced-3* mutant animals contained at least one refractile cell corpse (Table 4), indicating that these programmed cell deaths occur in animals lacking all *C. elegans* caspases. We observed caspase-independent cell corpses in different regions of the larval head, including positions internal and external to the pharynx, which suggests that multiple cell lineages – at low frequencies – generated caspase-independent cell corpses. Surprisingly, we discovered that engulfment-competent *ced-3* and *csp-3; csp-1; csp-2 ced-3* mutants also contained refractile cell corpses (Table 4). The number of cell corpses per *ced-3* or *csp-1; csp-2 ced-3* larva increased until 12 to 24 hours post hatching (see below; data not shown), indicating that at least some of the cell deaths occurred after embryogenesis. Given that all programmed cell deaths in the head normally occur embryonically and that cell corpses are never observed in the heads of wild-type larvae, we concluded that timing of cell deaths in these *ced-3* mutants was delayed. Thus, caspase-independent cell corpses can undergo an inefficient programmed cell death with slow kinetics in the absence of CED-3 activity, indicating that these cells likely die via CED-3-mediated apoptosis in wild-type animals.

### Caspase-independent cell corpses exhibit apoptotic morphology

**Figure 5:**
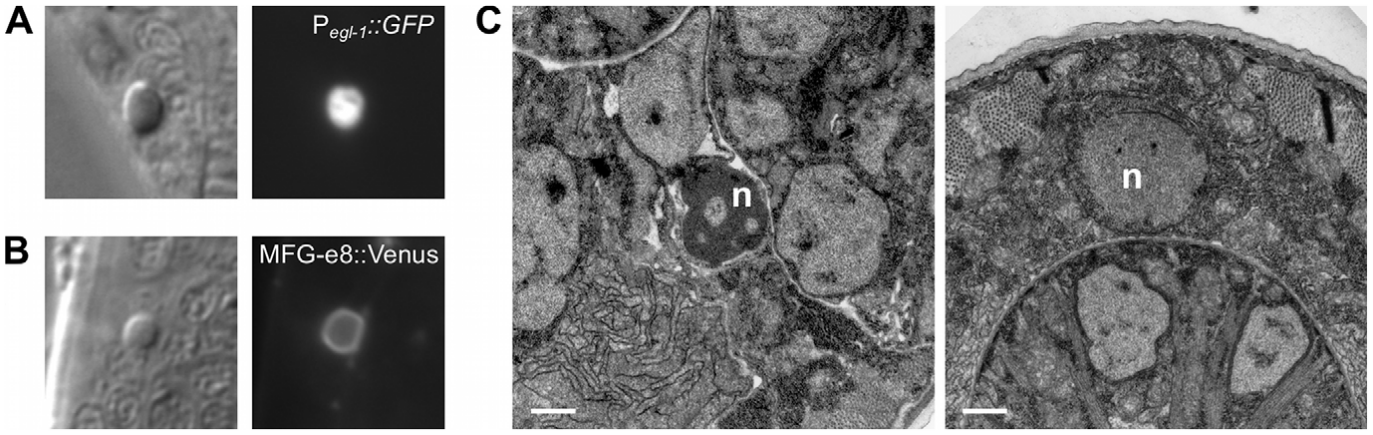
The cell corpses of caspase-deleted mutants are cytologically and morphologically apoptotic. (A) Nomarski DIC and fluorescence images of a cell corpse within the head of a *ced-1(e1735); csp-1(n4967); csp-2(n4871) ced-3(n3692)* L1 larva carrying the integrated transgene *nIs342[*P*_egl-1_::gfp]*, a transcriptional reporter that expresses GFP under the control of the BH3 domain-only encoding gene *egl-1*. (B) Nomarski DIC and fluorescence images of a cell corpse within the head of a *ced-1(e1735); csp-1(n4967); csp-2(n4871) ced-3(n3692)* L1 larva carrying the extrachromosomal array *nEx1646[*P*_dyn-1_::mfg-e8::Venus]*, a fusion protein that binds to cell-surface exposed phosphatidylserine. (C) Representative transmission electron micrographs of cell corpses from *ced-1(e1735); csp-1(n4967); csp-2(n4871) ced-3(n3692)* larvae 24 hrs post hatching. “n”, nuclei of the cell corpses; scale bars, 0.5 microns. Note the difference in chromatin condensation between the two cell corpses.

**Figure 6:**
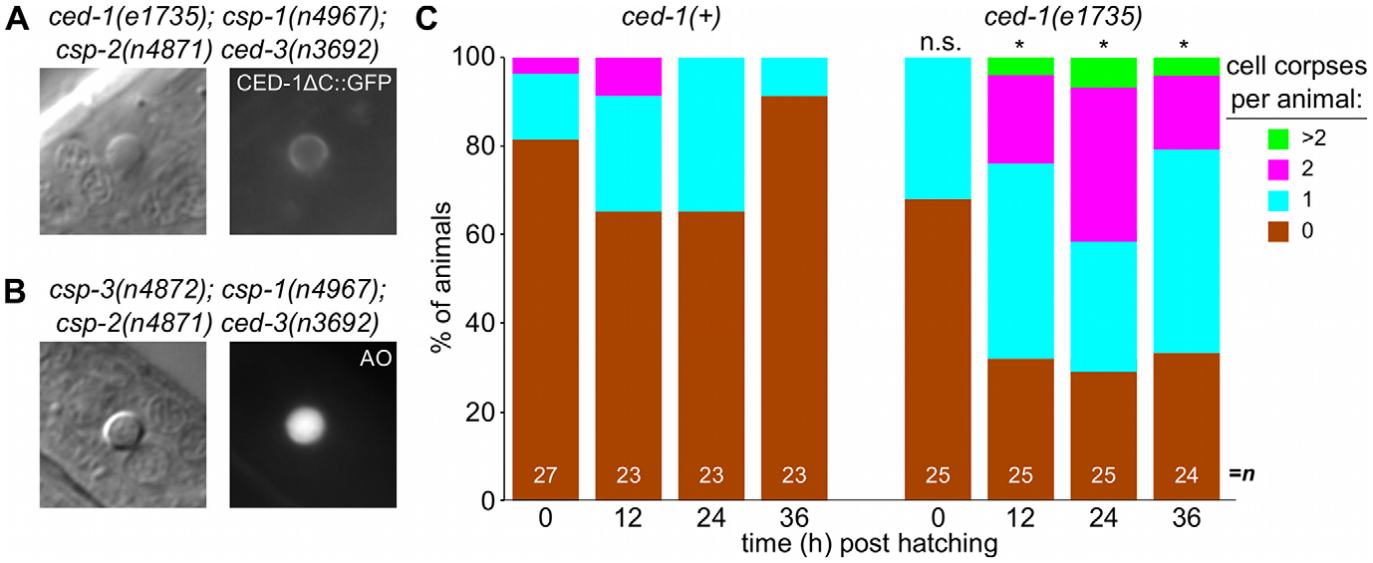
Caspase-independent cell corpses are engulfed and degraded. (A) Nomarski DIC and fluorescence images of a cell corpse from a *ced-1(e1735); csp-1(n4967); csp-2(n4871) ced-3(n3692)* L1 larva carrying the integrated transgene *nIs400[*P*_ced-1_::ced-1ΔC::gfp]*, which expresses a non-rescuing CED-1ΔC::GFP fusion protein. CED-1 is a transmembrane receptor that is expressed on engulfing cells, binds to apoptotic cell corpses, and is required for phagocytosis [46]. (B) Nomarski DIC and fluorescence images of a cell corpse from a *csp-3(n4872); csp-1(n4967); csp-2(n4871) ced-3(n3692)* L1 larva stained with acridine orange (AO), which fluoresces in engulfed cell corpses undergoing degradation in endosomal compartments. (C) The fraction of *csp-1(n4967); csp-2(n4871) ced-3(n3692)* and *ced-1(e1735); csp-1(n4967); csp-2(n4871) ced-3(n3692)* with 0, 1, 2 or >2 cell corpses at different time points post hatching. Asterisks indicate p<0.05 in a Mann-Whitney test comparing the two genotypes at a given time point.

Despite the strong causal link between caspase activation and apoptosis, recent studies have demonstrated that many morphological and biochemical changes associated with apoptosis can occur in the absence of caspases [4], [19], [21]. For example, in *C. elegans* the shed cells of *csp-3; csp-1; csp-2 ced-3* quadruple mutants exhibit phosphatidylserine exposure, expression of the pro-apoptotic BH3-only gene *egl-1*, and chromatin condensation [19]. To determine whether these apoptotic attributes are evident in caspase-independent programmed cell deaths that do not involve extrusion of the dying cell from the embryo, we characterized the cell corpses visible in caspase-deleted larvae (Figure 5 and Figure 6). In most of these experiments, we used strains with the wild-type *csp-3* allele, because (1) *csp-3* lacks a caspase active-site [15]; (2) although previous studies reported that *csp-3* has an anti-apoptotic function in somatic cells [22], we were unable to replicate those findings (Table S1); and, (3) the presence or absence of a *csp-3* mutation had no effect on the frequency or appearance of caspase-independent corpses (Table 4; Figure 6B; data not shown). Like *ced-3*-mediated programmed cell deaths in wild-type animals, the caspase-independent corpses expressed *egl-1*, the upstream activator of the canonical apoptosis pathway (Figure 5A). Also, these cell corpses displayed phosphatidylserine on their cell surfaces, as indicated by the phosphatidylserine-binding reporter MFG-e8::Venus (Figure 5B), and exhibited many of the morphological hallmarks of apoptosis, including contraction of cytoplasmic volume and, in some but not all cases, condensation of nuclear chromatin (Figure 5C).

Additionally, we noted that the caspase-independent cell corpses frequently stained with acridine orange (Figure 6A), suggesting that these corpses are engulfed, internalized and degraded via endosomal pathways, as are canonical programmed cell deaths [30], [48], [49], [51]. Indeed, we found that the caspase-independent corpses were recognized by CED-1 (Figure 6B), a receptor expressed on engulfing cells required for the efficient phagocytosis of cell corpses [46], [49], [50]. The recognition of caspase-independent cell corpses by CED-1 appeared to be functionally important, as *ced-1; csp-1; csp-2 ced-3* larvae contained more corpses than *csp-1; csp-2 ced-3* larvae (Figure 6C). Given that *ced-1* and other genes that function in cell-corpse engulfment promote programmed cell death [52], [53], it is unlikely that the *ced-1(e1735)* loss-of-function mutation caused additional cell deaths in the caspase-deleted mutants. Instead, the extra cell corpses in *ced-1* mutant larvae likely reflected an engulfment defect, consistent with the comparatively rapid degradation and disappearance of most caspase-independent corpses in *ced-1(+)* larvae within the 36-hour period after hatching (Figure 6C). We conclude that caspases are not required for programmed cell deaths to be recognized by the engulfment machinery, internalized and degraded. In short, many aspects of apoptosis, including phagocytosis – the ultimate fate of apoptotic cells – can occur without caspases. We conclude that a parallel, caspase-independent pathway contributes to programmed cell death in *C. elegans* and can execute most cellular changes associated with apoptosis.

## Discussion

Our experiments revealed unexpected complexities in the execution of apoptosis in *C. elegans*. While the CED-3 caspase is clearly the primary effector of programmed cell death, we demonstrated the existence of additional caspase-dependent and caspase-independent contributions to developmental apoptosis. Specifically, we observed that maternally-expressed caspase gene *csp-1* (but not *csp-2* or *csp-3*) promotes the deaths of a subset of cells programmed to die during *C. elegans* embryogenesis (Figure 1 and Figure 4; Table 1 and Table 3). Furthermore, ectopic expression of the *csp-1B* isoform of *csp-1* is sufficient to cell-autonomously kill cells that normally survive. These ectopic apoptotic cell deaths require the active site cysteine (C138) of CSP-1B, indicating that a caspase-like proteolytic function is responsible for its cell-killing activity (Table 2). The *C. elegans* genome therefore expresses at least two pro-apoptotic caspases, CED-3 and CSP-1B, to mediate programmed cell deaths. Nevertheless, the additional caspase activity conferred by *csp-1* cannot account for *ced-3*-independent programmed cell deaths that have been observed in *C. elegans*. For example, the non-apoptotic death of the male linker cell and the extrusion of shed cells were already known to be caspase-independent [18], [19]. Here we demonstrate that cells in caspase-deleted animals can undergo an apoptosis-like programmed cell death followed by engulfment, indicating that the complete apoptotic program can occur in the absence of caspases. Thus, in addition to CED-3 and CSP-1B, there are caspase-independent cell-killing activities that contribute to programmed cell deaths.

### CSP-1B is regulated by a mechanism distinct from that of CED-3

The caspases CED-3 and CSP-1B appear to be regulated differently. The auto-activation of CED-3 is facilitated by the Apaf-1 homolog CED-4 in a protein-protein interaction that requires the CED-3 prodomain [34]–[36]. In the absence of a pro-apoptotic signal, CED-9 sequesters CED-4 [37], thereby preventing its association with the inactive CED-3 proprotein. The CSP-1B proprotein lacks a long prodomain, suggesting that it is not activated through an association with the CED-4 octamer in cells undergoing apoptosis. Consistent with this expectation, we observed that the cell-killing activity of *csp-1B* transgenes, unlike that of *ced-3* transgenes, was not negatively regulated by *ced-9* (Figure 3). Furthermore, based on our genetic experiments (Figure 3) and the *in vitro* studies of Shaham [15], it does not appear that CSP-1B is activated by CED-3. We therefore propose that CSP-1B is regulated by a mechanism different from the canonical programmed cell death pathway that activates CED-3 and that CSP-1B likely promotes cell killing in parallel to CED-3 (Figure 3E).

There are no known or candidate regulators of *csp-1*. It is possible that *csp-1* is controlled entirely at the transcriptional level and that *csp-1* contributes a minor, sub-lethal pro-apoptotic activity to all cells within the *C. elegans* embryo. Indeed, only using sensitized backgrounds with partial defects in programmed cell death did we detect the pro-apoptotic function of *csp-1*. Nevertheless, we expect that it will be possible to identify regulators and effectors of *csp-1* through genetic screens for mutants that modify the cell-killing activity of *csp-1B* transgenes.

### Do the *csp* genes have non-apoptotic functions?

Given the minor contribution of *csp-1* to programmed cell death and the lack of a detectable role of *csp-2* or *csp-3* in apoptosis (Table 1; Table S1; data not shown), it is tempting to speculate that the *csp* genes have non-apoptotic functions in *C. elegans*. In *C. elegans*, *ced-3* functions in axon regeneration following laser axotomy [54]. In mammalian and *Drosophila* neurons, caspases have functions in dendritic pruning, axon guidance and the synaptic changes underlying long-term depression [14]. Caspase function is also required for the maturation of *Drosophila* sperm [55]. Interestingly, we observed robust expression of *csp-1* in the germlines of L4 and adult hermaphrodites, specifically in the late pachytene nuclei (Figure 4). We also observed temporally and spatially restricted *csp-2* and *csp-3* mRNA expression in the late pachytene nuclei of the L4 larval germline (data not shown), suggesting that the *csp* genes might have functions in germ cell development. However, mutant hermaphrodites and males carrying all tested combinations of *csp-1*, *csp-2* and *csp-3*, including the triple *csp* mutant were viable, fertile and failed to exhibit obvious brood-size defects that would suggest abnormalities in sperm or oocyte differentiation (data not shown).

### *csp-1B* as a tool for the genetic ablation of cells

Genetically encoded cell-killing activities provide an efficient and convenient method for determining cellular function through cell ablation. Killer genes such as *ced-3* have been used under the control of various promoters to ablate specific cells [32], [45], [56], [57]. However, the potent cell-killing activity of *ced-3* transgenes can cause organismic inviability, particularly if the promoter expression is not exclusive to a small number of cells (see below). *csp-1B* overexpression using the *mec-7* and *flp-15* promoters efficiently killed the touch and I2 neurons, respectively (Figure 2; Table 2; N. Bhatla and H.R. Horvitz, personal communication). The *mec-7* and *flp-15* promoters are relatively strong, as they also robustly induced *gfp* expression in these cells, such that the neural processes were visible with a dissecting microscope equipped with fluorescence optics. By contrast, the *odr-1* promoter did not produce detectable GFP expression in the neurites of the AWB, AWC and I1 neurons, and *csp-1B* under the control of the *odr-1* promoter failed to kill these cells even when injected at plasmid concentrations as high as 100 ng/µl (N. Bhatla and H.R. Horvitz, unpublished results). Thus, high levels of *csp-1B* expression might be required to kill most cells, making the use of *csp-1B* as a cell-ablation tool appropriate in situations in which the promoter sequence strongly drives expression in targeted cells and/or weakly promotes expression in additional cells not intended to be targets. For example, the P*_mec-7_::csp-1B* constructs, which were injected at a concentration of 15 ng/µl, produced *csp-1B* expression outside of the touch neurons that was detectable by fluorescence *in situ* hybridization. However, this level of *csp-1B* expression was sub-lethal and did not induce cell death or other cellular defects outside of the touch neurons (data not shown). By contrast, P*_mec-7_::ced-3* constructs were toxic to the animals when injected at concentrations above 1 ng/µl, suggesting that cells are very sensitive to ectopic *ced-3* and that using *ced-3* as a cell ablation tool is potentially problematic when promoter expression is not restricted to a small number of targeted cells.

### What is the role of *ced-3*-independent cell-killing activities that have minor contributions to programmed cell death?

Although the *csp-1* gene contributes a cell-killing activity to normal programmed cell deaths (Table 1), *csp-1* and the other *csp* genes are not responsible for the *ced-3*-independent programmed cell deaths present in the heads of *ced-3* larvae (Table 4). These deaths, like those of the male linker cell (ref. [18]; Table S5) and the embryonic shed cells [19], are caspase-independent – a surprising result in light of our observations that these cell corpses are morphologically apoptotic (Figure 5) and are engulfed (albeit with slower kinetics) like normal programmed cell deaths (Figure 6). Thus, the complete apoptotic program including cell-corpse internalization can occur in the absence of caspases in *C. elegans*, suggesting that the cellular changes accompanying apoptosis do not require proteolysis by the caspase family of proteases. Moreover, it is clear that apoptotic programmed cell deaths are achieved through the integration of independent cell-killing activities from CED-3, CSP-1B and an unknown caspase-independent source.

Given the minor cell-killing effects of the CSP-1B and the caspase-independent pathways, why might cell-killing activities in addition to that of CED-3 have evolved? It is possible that different cells, even within the set of *C. elegans* cells fated to die, are differentially sensitive to pro-apoptotic signals and that additional caspase and caspase-independent pathways ensure efficient and complete cell death under diverse environmental and developmental conditions. Interestingly, the postembryonic programmed cell deaths of the ventral cord are more sensitive to weak *ced-3* mutations than are the embryonic programmed cell deaths in the presumptive anterior pharynx: *ced-3* mutations that have weak effects in the anterior pharnyx typically have stronger effects in the ventral cord (ref. [17]; data not shown). We observed a complementary function for *csp-1*, which promotes apoptosis in the anterior pharynx (Table 1) but not in the ventral cord (Table S4).

In summary, multiple pro-apoptotic caspases function in programmed cell death in *C. elegans*, *Drosophila* and vertebrates. Furthermore, as we and others have shown, there are additional caspase-independent contributions to programmed cell deaths in *C. elegans*. We identified *C. elegans* caspase-independent cell deaths that are essentially identical to wild-type programmed cell deaths based on their apoptotic appearance and their recognition and internalization by engulfing cells. We expect that caspase-independent pro-apoptotic activities are present in other metazoans and that their identification will be of major importance to our understanding of cell death in the contexts of development and disease.

## Materials and Methods

### Strains

All *C. elegans* strains were cultured as described previously [58] and maintained at 20°C. We used Bristol N2 as the wild-type strain, and the mutations used in our experiments are listed below:

**LG I.** *unc-75(e950), ced-1(e1735), csp-3(n4872, tm2260, tm2286), nIs177[*P*_ceh-28_::gfp]* [59]

**LG II.** *csp-1(n4967, n5133, tm917), mab-10(n5117), lin-29(n836)*

**LG III.** *ced-4(n1162, n3158), ced-6(n2095), ced-7(n1996), ced-9(n1653, n2812), tat-1(tm1034), nIs308[*P*_mec-7_::csp-1B,* P*_mec-3_::gfp], nIs400[*P*_ced-1_::ced-1ΔC::gfp]* [19]

**LG IV.** *csp-2(n4871), ced-5(n1812), dpy-20(e1282), unc-30(e191), ced-3(n2427, n2436, n2452, n3692), nIs309[*P*_mec-7_::csp-1B,* P*_mec-3_::gfp]*

**LG V.** *egl-1(n1084 n3082), bcIs39[*P*_lim-7_::ced-1::gfp]* [45], *nIs342[*P*_egl-1_::4×NLS::gfp]* [59], *qIs56[*P*_lag-2_::gfp]*

**LG X.** *ced-8(n1891), bzIs8[*P*_mec-4_::gfp]* [22], *nIs106[*P*_lin-11_::gfp]* [52]

**Unknown linkage.** *nIs290[*P*_mec-3_::gfp]; nIs307[*P*_mec-7_::csp-1B,* P*_mec-3_::gfp], nIs368-370[*P*_mec-7_::csp-1B(C138S),* P*_mec-3_::gfp], nIs398[*P*_dyn-1_::mfg-e8::Venus]* [19], [60]

**Extrachromosomal arrays.** *nEx1646[*P*_dyn-1_::mfg-e8::Venus]* [19], [60], *nEx1465-71[csp-1(+) (pDD027)], nEx1604-9[csp-1B/C only (pDD030)], nEx1614-16[csp-1A only (pDD029)], nEx1617-19[csp-1-PD (pDD028)]*

### Plasmids

The P*_mec-7_::ced-3*, P*_mec-7_::ced-4* [32], P*_dyn-1_::mfg-e8::Venus* [60], P*_lim-7_::ced-1::gfp* [45], P*_ced-1_::ced-1ΔC::gfp* [46], P*_lin-11_::gfp* [52], P*_egl-1_::gfp* and P*_ceh-28_::gfp* [59] plasmids were described previously. The *csp-1* rescuing plasmid (pDD027) was constructed using PCR to amplify a 9 kb fragment of the *csp-1* genomic locus with the primers 5′-gtaacgccagggttttcccagtcacgacggtgatccttcggagcttcag and 5′- acgaggatatccgcattgag. The resulting amplicon was ligated via the TOPO-TA subcloning protocol into the pCR2.1 vector (Invitrogen). pDD028 (*csp-1-PD*), pDD029 (*csp-1A* only), and pDD030 (*csp-1B/C* only) were constructed using site-directed PCR mutagenesis. Two early stop codons in the *csp-1B/C* isoforms were generated in pDD028 using the primer 5′-ccgagaacggacgcctagtaatcgaaccataaac and its reverse complement. The *csp-1B/C* start codon was mutated to an alanine codon in pDD029 using the primer 5′-gactctcagagtcgagcgccgagaacggacgcc and its reverse-complement. Two early stop codons in the *csp-1A* isoform were generated in pDD030 using the primer 5′cctgaaaacgatagaagataattgataatcacaattcgacgatgatttgg and its reverse complement. The P*_mec-7_::csp-1A* plasmid (pDD003) was constructed using PCR to amplify the *csp-1A* cDNA from pDD006 using the primers 5′-gcggctagcatggtcctgaaaacgatagaag and 5′-gcgccatggttacatcgaccttgaaaagtgcc, which incorporate the restriction sites NheI and NcoI, respectively, into the resulting amplicon. The *csp-1A* amplicon was digested with NheI and NcoI and then ligated into the vector pPD52.102. The P*_mec-7_::csp-1B* plasmid (pDD002) was constructed by using PCR to amplify the *csp-1B* cDNA from pDD001 using the primers 5′-gcggctagcatgccgagaacggacgccaag and 5′-gcgccatggttacatcgaccttgaaaagtgcc, which incorporate the restriction sites NheI and NcoI, respectively. The *csp-1B* amplicon was digested with NheI and NcoI and then ligated into the vector pPD52.102, which encodes the *mec-7* promoter. The P*_mec-7_::csp-1B(C138S)* plasmid (pDD005) was constructed from pDD002 using PCR with the primers 5′-tggatgaactatacaaatagctgcgctccagcgcgttcgt and its reverse complement. The RNAi plasmid pL4440::*csp-1-PD* (pDD060) was constructed using PCR to amplify the prodomain encoding fragment of the *csp-1A* cDNA with the primers 5′-gcgagatctatggtcctgaaaacgatagaag and 5′-cgcctcgagatggcgggtttcagctgggtc, which incorporate the restriction sites BglII and XhoI, respectively. The resulting *csp-1-PD* amplicon was digested with BglII and XhoI and then ligated into pL4440. The RNAi plasmid pL4440::*csp-1B* (pDD061) was constructed using PCR to amplify the *csp-1B* cDNA with the primers 5′-gcgagatctatgccgagaacggacgccaag and 5′-cgcctcgagttacatcgaccttgaaaagtgcc, which incorporate the restriction sites BglII and XhoI, respectively. The resulting *csp-1B* amplicon was digested with BglII and XhoI and then ligated into pL4440.

### RNAi experiments

The *in vitro* transcription, purification, preparation and microinjection of *csp-1-PD* (pDD060) and *csp-1B* (pDD061) dsRNA were performed as described previously [61].

### Fluorescence *in situ* hybridization

The fixation of embryos and larval and adult animals, the conjugation of Cy5 or ALEXA594 fluorescent probes to *in situ* oligo probes, and the hybridization of oligos to fixed samples were performed as described previously [62]. All images were acquired using an inverted Nikon TE-2000 compound microscope equipped for fluorescence microscopy (Prior Scientific). Images were acquired with a PIXIS camera (Princeton Instruments) controlled by MetaMorph software (Molecular Devices) and modified for publication with ImageJ software (NIH). The “total *csp-1*” set of probes included 32 distinct 20-nucleotide sequences complementary to *csp-1B* (Biosearch Technologies, Inc). This set of oligos was conjugated to the fluorophore Cy5 (GE Healthcare) and hybridized to all three *csp-1* mRNA isoforms (*csp-1A*, *csp-1B* and *csp-1C*). The “*csp-1A*” set of probes included 32 distinct 20-nucleotide sequences complementary to the region of *csp-1A* that encodes the prodomain. This set of oligos was conjugated to the fluorophore ALEXA594 (Invitrogen) and hybridized specifically to the *csp-1A* mRNA isoform. Probe sequences are listed in Table S6.

### Cell-death assays and microscopy

The numbers of undead cells that failed to undergo programmed cell death in the anterior pharynges and postdeirid sensilla of L3 larvae were determined by direct observation using Nomarski optics as described previously [28]. Persistent cell corpses in larval heads also were quantified by direct observation using Nomarski optics; for this assay, larvae were staged by the time of hatching. For other cell-death assays, the ventral cord cells of young adults, the M4 neuron and its undead sister cell of L3 larvae, the touch neurons of L4 larvae, and the germ cell corpses of adult hermaphrodite gonads were identified using previously described GFP reporter transgenes [45], [52], [59]. For experiments involving ionizing radiation, L4 larvae were exposed to gamma irradiation from a Co-60 source. All strains were analyzed using a Zeiss Axioskop II compound microscope equipped for fluorescence microscopy. Images were acquired with an ORCA camera (Hammamatsu) controlled by OpenLab software (Perkin Elmer) and modified for publication using ImageJ (NIH).

### Transmission electron microscopy

L1-stage larvae were fixed, stained and sectioned for transmission electron microscopy as described previously [43]. Stained sections were imaged with a JEM-1200EX II microscope (JEOL) using an AMT XR41 CCD camera.

## Supporting Information

Figure S1 Transgenes that ectopically express *csp-1A* in the touch neurons lack cell-killing activity in both the presence and absence of the apoptosis regulator CED-9. The percentages of PLM cells that survive in strains carrying P*_mec-7_::csp-1A* transgenes. All strains contained the *ced-3(n3692)* mutation, which suppresses *ced-9(n2812)* inviability. n.s., *p*>0.05 in a Fisher's exact test.
(PDF)

Table S1 The deletion of *csp-1*, *csp-2* or *csp-3* does not cause the deaths of cells that normally survive. *(A)*. The touch neurons survive in *csp* mutants. The survival of AVM, ALML/R, PVM and PLML/R was scored using the transcriptional reporters *P_mec-3_::gfp* (*nIs290*)^a^ or *P_mec-4_::gfp* (*bzIs8*)^b^. *n*, animals scored. *(B)*. Mutants carrying *csp* deletions have the same number of pharyngeal cells as wild-type animals. The following pharyngeal cells were scored: the neurons I1, I2, I3, MC, MI, M3, M4 and NSM; the epithelial cells e1, e2, and e3; and, the muscle cells m1 and m2. In total, 34 cells were scored per pharynx. *n*, animals scored; SD, standard deviation.
(DOC)

Table S2 The defect in programmed cell death of *csp-1(n4967)* animals is rescued by transgenes that contain the endogenous *csp-1* promoter and coding regions. Mutations that alter the start of the B and C splicing isoforms of *csp-1* disrupt the rescuing activity of the *csp-1* transgene. The transgenes are described in detail in the legend of Figure 1 and in Materials and Methods. A Student's t-test was used to compare the *csp-1(n4967); ced-3(n2436)* strains with *csp-1* transgenes to the *csp-1(n4967); ced-3(n2436)* parental strain. *p* values were considered significant if less than 0.01 to correct for multiple comparisons.
(DOC)

Table S3 The deletion of *csp-2* or *csp-3* does not modify the defects in programmed cell death of *csp-1* and *ced-3* mutants. The average number of extra, undead cells in the pharynx was determined for each genotype. *n*, number of animals scored; SD, standard deviation. For the statistical comparisons between *ced-3(n2427)* or *ced-3(n2436)* and double mutants with each *csp* allele, *p* values were considered significant if less than 0.02 to correct for multiple comparisons.
(DOC)

Table S4 *csp-1* promotes the programmed cell death of (A) the M4 sister cell but not those of (B) the VC-like cells in the ventral cord or of (C) the V5.praap cell in the postdeirid sensillum. The survival of the M4 sister cell was scored using the integrated transgene *nIs177[*P*_ceh-28_::gfp]*. The number of extra VC-like cells was determined using the integrated transgene *nIs106[*P*_lin-11_::gfp]*. The survival of V5Rpaapp was determined via direct observation using Nomarski optics.
(DOC)

Table S5 The male linker cell dies in animals lacking all four caspases.
(DOC)

Table S6 Sequences of DNA probes used for fluoresence *in situ* hybridization (FISH) experiments. The *csp-1A* oligos hybridize to the region of *csp-1A* that encodes the prodomain and are therefore specific to the *csp-1A* isoform. The “total” *csp-1* oligos hybridize to a region present in all known *csp-1* mRNA isoforms.
(DOCX)
