# Supplementary figures and images for: Both the Caspase CSP-1 and a Caspase-Independent Pathway Promote Programmed Cell Death in Parallel to the Canonical Pathway for Apoptosis in Caenorhabditis elegans
Source: PLoS Genet. 2013 Mar 7;9(3):e1003341. doi: 10.1371/journal.pgen.1003341 (PMC3591282; doi:10.1371/journal.pgen.1003341)

# Figure S1

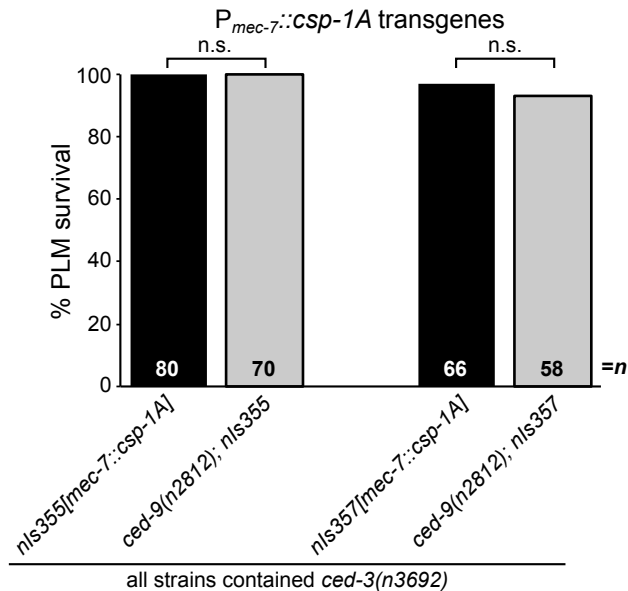

Supplement: Figure S1 — Transgenes that ectopically express csp-1A in the touch neurons lack cell-killing activity in both the presence and absence of the apoptosis regulator CED-9. The percentages of PLM cells that survive in strains carrying Pmec-7::csp-1A transgenes. All strains contained the ced-3(n3692) mutation, which suppresses ced-9(n2812) inviability. n.s., p>0.05 in a Fisher's exact test. (PDF) [file pgen.1003341.s001.pdf]
