# Supplementary material for: Both the Caspase CSP-1 and a Caspase-Independent Pathway Promote Programmed Cell Death in Parallel to the Canonical Pathway for Apoptosis in Caenorhabditis elegans
Source: PLoS Genet. 2013 Mar 7;9(3):e1003341. doi: 10.1371/journal.pgen.1003341 (PMC3591282; doi:10.1371/journal.pgen.1003341)
Supplement: Table S1 — The deletion of csp-1, csp-2 or csp-3 does not cause the deaths of cells that normally survive. (A). The touch neurons survive in csp mutants. The survival of AVM, ALML/R, PVM and PLML/R was scored using the transcriptional reporters Pmec-3::gfp (nIs290)a or Pmec-4::gfp (bzIs8)b. n, animals scored. (B). Mutants carrying csp deletions have the same number of pharyngeal cells as wild-type animals. The following pharyngeal cells were scored: the neurons I1, I2, I3, MC, MI, M3, M4 and NSM; the epithelial cells e1, e2, and e3; and, the muscle cells m1 and m2. In total, 34 cells were scored per pharynx. n, animals scored; SD, standard deviation. (DOC) [file pgen.1003341.s002.doc]

**Table S1**. The deletion of *csp-1*, *csp-2* or *csp-3* does not cause the deaths of cells that normally survive.

*A*. The touch neurons survive in *csp* mutants. The survival of AVM, ALML/R, PVM and PLML/R was scored using the transcriptional reporters *P_mec-3_::gfp* (*nIs290*)^a^ or *P_mec-4_::gfp* (*bzIs8*)^b^. *n*, animals scored.

|  |  | % survival | | | | |
| --- | --- | --- | --- | --- | --- | --- |
| genotype | *n* | | AVM | ALML/R | PVM | PLML/R |
| *nIs290*  *bzIs8* | 54  66 | | 100  100 | 100  100 | 100  100 | 100  98 |
| *csp-1(n4967)*^a^ | 30 | | 100 | 100 | 100 | 100 |
| *csp-2(n4871)* ^a^ | 30 | | 100 | 100 | 100 | 100 |
| *csp-3(n4872)* ^a^ | 59 | | 100 | 100 | 100 | 100 |
| *csp-3(tm2260)*^b^ | 70 | | 100 | 99 | 100 | 99 |
| *csp-3(tm2486)* ^b^ | 75 | | 100 | 97 | 100 | 96 |
| *csp-3(n4872); csp-2(n4871)* ^a^ | 43 | | 100 | 100 | 100 | 100 |
| *ced-9(n2812)* ^b§^ | 28 | | 86 | 96 | 64 | 18 |
| *tat-1(tm1034)*^b^ | 44 | | 100 | 100 | 100 | 100 |

^§^*ced-9(n2812)* homozygous animals were the progeny of *ced-9(n2812)/qC1* heterozygotes.

*B.* Mutants carrying *csp* deletions have the same number of pharyngeal cells as wild-type animals. The following pharyngeal cells were scored: the neurons I1, I2, I3, MC, MI, M3, M4 and NSM; the epithelial cells e1, e2, and e3; and, the muscle cells m1 and m2. In total, 34 cells were scored per pharynx. *n*, animals scored; SD, standard deviation.

| genotype | missing cells per pharynx ± SD | *n* |
| --- | --- | --- |
| wild-type | 0.0 ± 0.0 | 14 |
| *csp-1(n4967)* | 0.0 ± 0.0 | 17 |
| *csp-2(n4871)* | 0.0 ± 0.0 | 18 |
| *csp-3(n4872)* | 0.0 ± 0.0 | 18 |
| *csp-3(tm2260)* | 0.0 ± 0.0 | 11 |
| *csp-3(tm2486)* | 0.1 ± 0.3 | 15 |
| *ced-9(n1653ts)* 20°C | 0.8 ± 1.1 | 18 |
| *ced-9(n1653ts)* 22.5°C | 0.8 ± 1.1 | 20 |
| *ced-9(n1653ts)* 25°C | 1.1 ± 1.3 | 13 |
