# Supplementary material for: Both the Caspase CSP-1 and a Caspase-Independent Pathway Promote Programmed Cell Death in Parallel to the Canonical Pathway for Apoptosis in Caenorhabditis elegans
Source: PLoS Genet. 2013 Mar 7;9(3):e1003341. doi: 10.1371/journal.pgen.1003341 (PMC3591282; doi:10.1371/journal.pgen.1003341)
Supplement: Table S2 — The defect in programmed cell death of csp-1(n4967) animals is rescued by transgenes that contain the endogenous csp-1 promoter and coding regions. Mutations that alter the start of the B and C splicing isoforms of csp-1 disrupt the rescuing activity of the csp-1 transgene. The transgenes are described in detail in the legend of Figure 1 and in Materials and Methods. A Student's t-test was used to compare the csp-1(n4967); ced-3(n2436) strains with csp-1 transgenes to the csp-1(n4967); ced-3(n2436) parental strain. p values were considered significant if less than 0.01 to correct for multiple comparisons. (DOC) [file pgen.1003341.s003.doc]

**Table S2.** The defect in programmed cell death of *csp-1(n4967)* animals is rescued by transgenes that contain the endogenous *csp-1* promoter and coding regions. Mutations that alter the start of the B and C splicing isoforms of *csp-1* disrupt the rescuing activity of the *csp-1* transgene. The transgenes are described in detail in the legend of Figure 1 and in Materials and Methods. A Student’s t-test was used to compare the *csp-1(n4967); ced-3(n2436)* strains with *csp-1* transgenes to the *csp-1(n4967); ced-3(n2436)* parental strain. *p* values were considered significant if less than 0.01 to correct for multiple comparisons.

| genotype | no. extra cells  per anterior pharynx ± SD | *n* | *p* value |
| --- | --- | --- | --- |
| *ced-3(n2436)* | 6.2 ± 1.4 | 37 | - |
| *csp-1(n4967); ced-3(n2436)* | 8.6 ± 1.6 | 29 | **<0.001** |
|  |  |  |  |
| *csp-1; ced-3(n2436); Ex[csp-1]* #1 | 6.1 ± 1.2 | 16 | **<0.001** |
| *csp-1; ced-3(n2436); Ex[csp-1]* #2 | 4.9 ± 1.8 | 14 | **<0.001** |
| *csp-1; ced-3(n2436); Ex[csp-1]* #3 | 7.0 ± 1.8 | 13 | 0.024 |
| *csp-1; ced-3(n2436); Ex[csp-1]* #4 | 7.1 ± 0.7 | 7 | **0.002** |
|  |  |  |  |
| *csp-1; ced-3(n2436); Ex[csp-1 PD only] #1* | 8.6 ± 1.4 | 14 | n.s. |
| *csp-1; ced-3(n2436); Ex[csp-1 PD only] #2* | 8.8 ± 1.5 | 15 | n.s. |
| *csp-1; ced-3(n2436); Ex[csp-1 PD only] #3* | 9.0 ± 1.9 | 15 | n.s. |
| *csp-1; ced-3(n2436); Ex[csp-1 PD only] #4* | 8.4 ± 1.5 | 16 | n.s. |
| *csp-1; ced-3(n2436); Ex[csp-1 PD only] #5* | 8.8 ± 1.4 | 15 | n.s. |
| *csp-1; ced-3(n2436); Ex[csp-1 PD only] #6* | 9.1 ± 1.6 | 15 | n.s. |
| *csp-1; ced-3(n2436); Ex[csp-1 PD only] #7* | 8.3 ± 2.4 | 15 | n.s. |
|  |  |  |  |
| *csp-1; ced-3(n2436); Ex[csp-1A only] #1* | 8.7 ± 1.9 | 18 | n.s. |
| *csp-1; ced-3(n2436); Ex[csp-1A only] #2* | 8.1 ± 1.7 | 15 | n.s. |
| *csp-1; ced-3(n2436); Ex[csp-1A only] #3* | 8.1 ± 2.0 | 15 | n.s. |
| *csp-1; ced-3(n2436); Ex[csp-1A only] #4* | 8.3 ± 1.3 | 16 | n.s. |
| *csp-1; ced-3(n2436); Ex[csp-1A only] #5* | 8.1 ± 2.5 | 16 | n.s. |
|  |  |  |  |
| *csp-1; ced-3(n2436); Ex[csp-1B/C only] #1* | 6.6 ± 1.9 | 16 | **0.001** |
| *csp-1; ced-3(n2436); Ex[csp-1B/C only] #2* | 6.3 ± 1.4 | 15 | **<0.001** |
| *csp-1; ced-3(n2436); Ex[csp-1B/C only] #3* | 6.9 ± 3.2 | 15 | n.s. |
| *csp-1; ced-3(n2436); Ex[csp-1B/C only] #4* | 6.3 ± 2.3 | 14 | **0.003** |
| *csp-1; ced-3(n2436); Ex[csp-1B/C only] #5* | 6.3 ± 2.5 | 15 | **0.005** |
| *csp-1; ced-3(n2436); Ex[csp-1B/C only] #6* | 7.6 ± 1.9 | 14 | n.s. |

A Student’s t-test was used to compare the *csp-1(n4967); ced-3(n2436)* strains with *csp-1* transgenes to the *csp-1(n4967); ced-3(n2436)* parental strain. *p* values were considered significant if less than 0.01 to correct for multiple comparisons.
