# Supplementary material for: Both the Caspase CSP-1 and a Caspase-Independent Pathway Promote Programmed Cell Death in Parallel to the Canonical Pathway for Apoptosis in Caenorhabditis elegans
Source: PLoS Genet. 2013 Mar 7;9(3):e1003341. doi: 10.1371/journal.pgen.1003341 (PMC3591282; doi:10.1371/journal.pgen.1003341)
Supplement: Table S3 — The deletion of csp-2 or csp-3 does not modify the defects in programmed cell death of csp-1 and ced-3 mutants. The average number of extra, undead cells in the pharynx was determined for each genotype. n, number of animals scored; SD, standard deviation. For the statistical comparisons between ced-3(n2427) or ced-3(n2436) and double mutants with each csp allele, p values were considered significant if less than 0.02 to correct for multiple comparisons. (DOC) [file pgen.1003341.s004.doc]

**Table S3.** The deletion of *csp-2* or *csp-3* does not modify the defects in programmed cell death of *csp-1* and *ced-3* mutants. The average number of extra, undead cells in the pharynx was determined for each genotype. *n*, number of animals scored; SD, standard deviation. For the statistical comparisons between *ced-3(n2427)* or *ced-3(n2436)* and double mutants with each *csp* allele, *p* values were considered significant if less than 0.02 to correct for multiple comparisons.

| genotype | extra cells per  anterior pharynx ± SD | *n* | *p* value |
| --- | --- | --- | --- |
| *csp-1(n4967)* | 0.3 ± 0.4 | 16 | - |
| *csp-1(n4967); csp-2(n4871)* | 0.3 ± 0.4 | 16 | n.s. |
| *csp-3(n4872); csp-1(n4967)* | 0.1 ± 0.3 | 16 | n.s. |
|  |  |  |  |
| *csp-1(n4967); ced-3(n2427)* | 3.0 ± 1.3 | 38 | - |
| *csp-1(n4967); csp-2 (n4871) ced-3(n2427)* | 3.2 ± 0.9 | 17 | n.s. |
| *csp-3(n4872); csp-1(n4967); ced-3(n2427)* | 2.9 ± 1.0 | 18 | n.s. |
|  |  |  |  |
| *csp-1(n4967); ced-3(n2436)* | 8.6 ± 1.6 | 29 | - |
| *csp-1(n4967); csp-2 (n4871) ced-3(n2436)* | 7.9 ± 1.3 | 17 | n.s. |
| *csp-3(n4872); csp-1(n4967); ced-3(n2436)* | 8.3 ± 2.0 | 18 | n.s. |

For the statistical comparisons between *ced-3(n2427)* or *ced-3(n2436)* and double mutants with each *csp* allele, *p* values were considered significant if less than 0.02 to correct for multiple comparisons.
