# Supplementary material for: Both the Caspase CSP-1 and a Caspase-Independent Pathway Promote Programmed Cell Death in Parallel to the Canonical Pathway for Apoptosis in Caenorhabditis elegans
Source: PLoS Genet. 2013 Mar 7;9(3):e1003341. doi: 10.1371/journal.pgen.1003341 (PMC3591282; doi:10.1371/journal.pgen.1003341)
Supplement: Table S4 — csp-1 promotes the programmed cell death of (A) the M4 sister cell but not those of (B) the VC-like cells in the ventral cord or of (C) the V5.praap cell in the postdeirid sensillum. The survival of the M4 sister cell was scored using the integrated transgene nIs177[Pceh-28::gfp]. The number of extra VC-like cells was determined using the integrated transgene nIs106[Plin-11::gfp]. The survival of V5Rpaapp was determined via direct observation using Nomarski optics. (DOC) [file pgen.1003341.s005.doc]

**Table S4.** *csp-1* promotes the programmed cell death of (A) the M4 sister cell but not those of (B) the VC-like cells in the ventral cord or of (C) the V5.praap cell in the postdeirid sensillum. The survival of the M4 sister cell was scored using the integrated transgene *nIs177[*P*_ceh-28_::gfp]*. The number of extra VC-like cells was determined using the integrated transgene *nIs106[*P*_lin-11_::gfp]*. The survival of V5Rpaapp was determined via direct observation using Nomarski optics.

Part A.

| genotype | % survival of the  M4 sister cell | *n* | *p* value |
| --- | --- | --- | --- |
| wild-type | 1 | 162 | - |
| *csp-1(n4967)* | 1 | 210 | n.s. |
|  |  |  |  |
| *ced-3(n2427)* | 35 | 210 | - |
| *csp-1(n4967); ced-3(n2427)* | 61 | 340 | <0.0001 |
|  |  |  |  |
| *ced-3(n2436)* | 70 | 263 | - |
| *csp-1(n4967); ced-3(n2436)* | 81 | 348 | 0.002 |

Part B.

| genotype | extra VC-like cells  per ventral cord ± SD | *n* | *p* value |
| --- | --- | --- | --- |
| wild-type | 0.0 ± 0.1 | 45 | - |
| *csp-1(n4967)* | 0.0 ± 0.0 | 30 | n.s. |
|  |  |  |  |
| *ced-3(n2427)* | 1.7 ± 1.3 | 90 | - |
| *csp-1(n4967); ced-3(n2427)* | 1.6 ± 1.0 | 48 | n.s. |

Part C.

| genotype | % survival of  V5Rpaapp | *n* | *p* value |
| --- | --- | --- | --- |
| wild-type | 0 | 25 | - |
| *csp-1(n4967)* | 4 | 26 | n.s. |
|  |  |  |  |
| *ced-3(n2427)* | 0 | 27 | - |
| *csp-1(n4967); ced-3(n2427)* | 0 | 25 | n.s. |
|  |  |  |  |
| *ced-3(n2436)* | 50 | 26 | - |
| *csp-1(n4967); ced-3(n2436)* | 52 | 23 | n.s. |
