# Supplementary material for: Both the Caspase CSP-1 and a Caspase-Independent Pathway Promote Programmed Cell Death in Parallel to the Canonical Pathway for Apoptosis in Caenorhabditis elegans
Source: PLoS Genet. 2013 Mar 7;9(3):e1003341. doi: 10.1371/journal.pgen.1003341 (PMC3591282; doi:10.1371/journal.pgen.1003341)
Supplement: Table S5 — The male linker cell dies in animals lacking all four caspases. (DOC) [file pgen.1003341.s006.doc]

**Table S5**. The male linker cell dies in animals lacking all four caspases.

| genotype | % survival of  the linker cell | *n* | *p* value |
| --- | --- | --- | --- |
| wild-type^a,b^ | 0 | 27 | - |
| *lin-29(n836)^a,b^* | 66 | 32 | <0.0001 |
| *mab-10(n5117)^a,b^* | 42 | 31 | <0.0001 |
| *csp-3(n4872); csp-1(n4967); csp-2(n4871) ced-3(n3692)^c^* | 0 | 27 | n.s. |

^a^Homozygous for *him-8(e1489)*.

^b^Homozygous for *qIs56[*P*_lag-2_::gfp]*.

^c^Heterozygous for *qIs56/+*.
