# Supplementary material for: Both the Caspase CSP-1 and a Caspase-Independent Pathway Promote Programmed Cell Death in Parallel to the Canonical Pathway for Apoptosis in Caenorhabditis elegans
Source: PLoS Genet. 2013 Mar 7;9(3):e1003341. doi: 10.1371/journal.pgen.1003341 (PMC3591282; doi:10.1371/journal.pgen.1003341)
Supplement: Table S6 — Sequences of DNA probes used for fluoresence in situ hybridization (FISH) experiments. The csp-1A oligos hybridize to the region of csp-1A that encodes the prodomain and are therefore specific to the csp-1A isoform. The “total” csp-1 oligos hybridize to a region present in all known csp-1 mRNA isoforms. (DOCX) [file pgen.1003341.s007.docx]

**Table S6**. Sequences of DNA probes used for fluoresence *in situ* hybridization (FISH) experiments. The *csp-1A* oligos hybridize to the region of *csp-1A* that encodes the prodomain and are therefore specific to the *csp-1A* isoform. The “total” *csp-1* oligos hybridize to a region present in all known *csp-1* mRNA isoforms.

| *csp-1A* oligos | “total” *csp-1* oligos |
| --- | --- |
| \| cttctatcgttttcaggacc \| \| --- \| \| cgtcgaattgtgatttacaa \| \| ttgaaatcttcgaccaaatc \| \| gacatagaagaagtcgtttg \| \| agtagatattgttgttgatg \| \| gttgactcgatttcgatcgt \| \| gccacttctgcatatttcga \| \| ccaagaattggctcttctgt \| \| ggaatttcagaaatcgttcc \| \| ctacttgttttgttttgtct \| \| ccttttgggagaaaggttct \| \| cgtcgttggaaaactgcata \| \| gtttcaatgcagagctcaga \| \| ggagtggttggaactttttc \| \| catgtttgaattgcattggg \| \| catagtttcctcgtcgaact \| \| cctgcaccatagatcattct \| \| ccagaaatccacagagtcta \| \| ttgtcgctttcgatgtgaac \| \| cttgagtagcagtctagtga \| \| gtcggaagagtatgagatca \| \| gagtaccacttaaggtcaga \| \| tgatatgtgaatccagaacg \| \| tgataagtaacagggggtgt \| \| tggatccttattttcagtcc \| \| ctggagatggctcttgtata \| \| ctcgattgtacatctagaaa \| \| tggtttcatacttgaaccca \| \| tggtgggcttatcaagtatc \| \| gtttcagctgggtcatctag \| \| agtgaatacgagcagtcatg \| \| actctgagagtcatactcct \| | \| atggttcgatttcttggcgt \| \| --- \| \| ctcggatttgaattcatctc \| \| acttaatatgaggacggtcc \| \| ccatattcttgaagttctcg \| \| tgtttcgttccgactcttct \| \| cttggttagatttacttcgt \| \| ctgtgtactgaagcttttgg \| \| gcttcaagattccgtttgca \| \| gatagcttcaagcatgcttt \| \| atgtgccatctccgcgaatt \| \| ggaagagtatgatcgaatcg \| \| gctccatcaccatgacttaa \| \| gtcatcaattccaaagacac \| \| acctccattacattgacagg \| \| atgatacgctaaatatgtgg \| \| catttgggcttgagaagaag \| \| attttcctccgcgacatgct \| \| tcaacgggaacacccatgtt \| \| tttgtcttccaaagctggca \| \| caaaatttcgagattggagc \| \| cggcattatacgtgacatca \| \| gcgttcaatgaagtgaaggt \| \| ggaaaacgagatgatgacgt \| \| tcgataagacgtaaacccgt \| \| aatataccatgtgcccgctt \| \| tgaagactttacacatggat \| \| ggtgcatagtttttgaatgc \| \| catttctaccagtttccgtt \| \| cgttcccttgaacattctca \| \| tctggggcttgttttaatac \| \| gtttggtgagcctggataat \| \| atcgaccttgaaaagtgcca \| |
